# Racial and Ethnic Disparities in Care for Pediatric Sleep‐Disordered Breathing

**DOI:** 10.1002/oto2.70246

**Published:** 2026-05-28

**Authors:** Colleen C. McLaughlin, Jesse L. Hawke, Emily F. Boss, Guy M. Brock, Meredith M. Lind, Tasleem J. Padamsee, Prasanth Pattisapu, Deena J. Chisolm, Laura J. Chavez

**Affiliations:** ^1^ Center for Child Health Equity and Outcomes Research, Abigail Wexner Research Institute at Nationwide Children's Hospital Columbus Ohio USA; ^2^ Department of Otolaryngology Johns Hopkins University School of Medicine Baltimore Maryland USA; ^3^ Department of Biomedical Informatics and Center for Biostatistics Ohio State University Columbus Ohio USA; ^4^ Department of Otolaryngology Ohio State University and Nationwide Children's Hospital Columbus Ohio USA; ^5^ Division of Health Services Management and Policy, College of Public Health Ohio State University Columbus Ohio USA; ^6^ Department of Pediatrics, College of Medicine Ohio State University Columbus Ohio USA

**Keywords:** ethnic and racial minorities, healthcare disparities, pediatrics, polysomnography, sleep‐disordered breathing, tonsillectomy

## Abstract

**Objective:**

Obstructive sleep disordered breathing is the most common indication for pediatric tonsillectomy in the United States, but there are barriers to specialty care that may contribute to disparities in tonsillectomy use. This study examined the association between race, ethnicity, urban/rural residence, and other factors in access to specialty care for children diagnosed with obstructive sleep disordered breathing.

**Study Design:**

Retrospective observational cohort study.

**Setting:**

Fifteen states participating in the United States Medicaid and Child Health Insurance Programs.

**Methods:**

Children ages 2 to 18 years from 2017 to 2018 with a diagnosis of obstructive sleep disordered breathing followed for 1 year to ascertain receipt of polysomnography, otolaryngology specialist visit, and tonsillectomy.

**Results:**

This study included 304,415 children with diagnosis of obstructive sleep disordered breathing. Care pathways differed by race and ethnicity; Black non‐Hispanic and Hispanic children were more likely to undergo polysomnography, either alone or with other forms of care (adjusted rate ratios and 95% confidence intervals 1.06 [1.04‐1.09] and 1.06 [1.04‐1.08], respectively). White non‐Hispanic children, on the other hand, were more likely to receive otolaryngology care and/or tonsillectomy without polysomnography (Black non‐Hispanic: 0.97 [0.95‐0.99], Hispanic: 0.93 [0.92‐0.95]). Urban children were more likely to undergo polysomnography alone and polysomnography prior to otolaryngology care (Urban: 1.06 [1.03‐1.09], relative to rural).

**Conclusion:**

Black non‐Hispanic, Hispanic, and urban children were more likely to undergo polysomnography and less likely to have tonsillectomy in absence of polysomnography. These differences suggest potential unwarranted variation in diagnostic and surgical care across race/ethnic groups and urban/rural settings.

Pediatric obstructive sleep disordered breathing (oSDB), which ranges from habitual snoring to obstructive sleep apnea syndrome (OSAS), affects over 10% of children.^1^, ^2^, ^3^, ^4^, ^5^ Poor sleep quality arising from oSDB is associated with adverse neurobehavioral and neurocognitive outcomes, including depression and poor school performance.^6^, ^7^, ^8^, ^9^ While surgery with adenotonsillectomy (hereafter tonsillectomy) is the definitive treatment for OSAS in children with adenotonsillar hypertrophy, tonsillectomy use is a source of health inequity in high‐income countries including the United States (US).^10^, ^11^, ^12^, ^13^, ^14^, ^15^, ^16^, ^17^, ^18^ In the US, tonsillectomy is more common among White non‐Hispanic (hereafter referred to as White) children, children living in rural settings, and children with public insurance.^10^, ^11^, ^12^, ^15^, ^16^, ^17^, ^18^, ^19^, ^20^, ^21^, ^22^, ^23^, ^24^, ^25^, ^26^, ^27^, ^28^ This variation exceeds what would be expected based on underlying health status, given children of color and children with socioeconomic disadvantage have elevated risk of OSAS.^2^, ^4^, ^5^, ^15^, ^29^, ^30^, ^31^, ^32^

While the unwarranted variation in tonsillectomy has been observed for almost a century,^33^, ^34^, ^35^, ^36^ research regarding variation in other forms of oSDB care by race‐ethnicity is more recent and limited. Three claims‐based studies have examined care pathways among children with a diagnosis of oSDB.^10^, ^11^, ^12^ In these studies of Medicaid‐insured children, Black non‐Hispanic (Black) and Hispanic children had lower rates of oSDB‐directed care, higher rates of PSG, and lower rates of tonsillectomy without PSG, relative to White children. However, these studies^10^, ^11^, ^12^ lacked geolocation data and so were unable to adjust for geographic differences in the availability of PSG, which has been cited as a factor in the decision to have surgery without polysomnographic confirmation of disease.^37^, ^38^, ^39^

This study builds on this prior work by examining care for children with a diagnosis of oSDB in a larger sample of children enrolled in US Medicaid and Children's Health Insurance Program (CHIP) across more states (15 states 2017‐2018). Our aim was to describe variation in the care patterns for children with oSDB by race‐ethnicity and urban/rural residence. The availability of ZIP Codes in our data allowed for calculation of local estimates of PSG and tonsillectomy rates among Medicaid children in included states, which can provide a proxy for geographic variation in availability. Adjusting for local treatment rates helps to ensure the observed associations between race/ethnicity or urban/rural residence and care pathways are not attributable to differences in service availability.

## Methods

### Study Population

This study used the Transformed Medicaid Statistical Information System (T‐MSIS) analytic files and included Medicaid/CHIP enrollees ages 2 to 18 years from 2017 to 2018 from 15 US States (California, Delaware, Florida, Indiana, Kentucky, Maine, Nevada, New Hampshire, North Carolina, Ohio, Pennsylvania, South Dakota, Texas, Washington).^40^ These states were identified as having high or moderate usability of race‐ethnicity data.^41^ T‐MSIS data for 2016 through 2019 were used to capture medical history and follow‐up care.

Beneficiaries were included if they had a new diagnosis of oSDB (index visit) between 2017 and 2018 (Supplemental Table S1, available online contains coding criteria). To capture both medical history and follow‐up care, beneficiaries had to have been enrolled in Medicaid (not dual eligible) at least 11 out of 12 months before and after their index visit. Beneficiaries were excluded if they had missing race‐ethnicity, sex, or ZIP Code. To increase the specificity of oSDB as the indication for care follow‐up, we excluded beneficiaries with 12‐month clinical history of otolaryngological specialty care, medical complexities affecting oSDB care (Down syndrome, craniofacial abnormalities, neuromuscular disorders, sickle cell disease, or mucopolysaccharidoses), or 5 or more throat infections.^37^ Because of relatively small numbers and concerns about data quality, beneficiaries who had race‐ethnicity other than Black, Hispanic, or White were excluded.

### Outcomes

**Figure 1:**
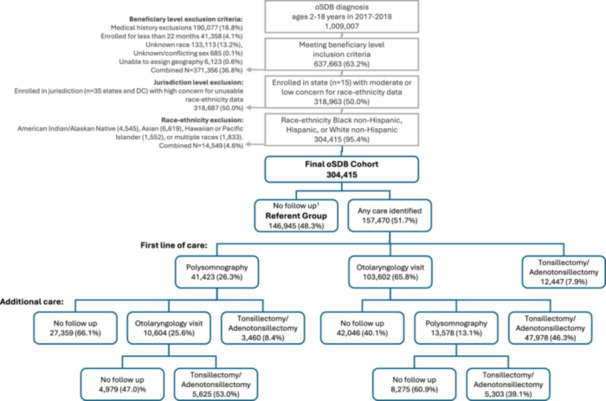
CONSORT diagram and all measured care pathways for obstructive sleep disordered breathing, Medicaid beneficiaries ages 2 to 18 years, 2017 to 2018, 15 States.

The outcomes of interest were overnight PSG, otolaryngology specialist visit, and tonsillectomy/adenotonsillectomy.^37^ To identify tonsillectomy, we used claims within 1 year of the index visit. To identify PSG and otolaryngology, we used claims from 90 days before the index visit through the date of tonsillectomy or 365 days, whichever came first. We report use of PSG, otolaryngology, and tonsillectomy measured independently (ie, not mutually exclusive categories), using children with no evidence of oSDB‐directed care as the comparison group. In addition, 8 care pathways were defined based on mutually exclusive combinations of the 3 measured forms of care relative to no evidence of oSDB‐directed care (Figure 1). We also examined the timing of first PSG claim in relation to the first otolaryngology claim.

### Independent Variables

Medicaid enrollment files were used to determine race/ethnicity, age, sex, and ZIP code of residence. Urban/rural was based on the 2010 Rural/Urban Commuting Area codes for ZIP Codes.^42^ Metropolitan areas plus all other ZIP Codes with greater than 30% secondary flow to metropolitan areas were designated as urban, all other valid ZIP Codes were considered rural.

The Medicaid outpatient and inpatient claims in the 12 months prior to the index visit were used to identify obesity, asthma, attention deficit/hyperactivity disorder (ADHD), autism spectrum disorder, number of throat infections, attendance at a well child visit, and emergency department or urgent care (ED/UC) reliance. ED/UC reliance was defined as >33% of outpatient visits at emergency department or urgent care, excluding ED resulting in admission.

We used all enrolled beneficiaries ages 0 to 18 years to calculate local PSG and tonsillectomy rates from 2017 to 2019 for aggregate groupings of ZIP Codes approximating the US Census Public Use Microdata Areas (PUMAs).^43^ PUMAs are nonoverlapping contiguous geographic areas with no fewer than 100,000 residents and can range from neighborhoods within cities to multiple counties in rural areas.

### Statistical Analysis

We used multivariable Poisson regression with robust error variance estimators to derive the rate ratios (RR) and 95% confidence intervals (CI) for each care pathway relative to no evidence of oSDB‐directed care. Models included age, sex, race/ethnicity, rurality, obesity, asthma, autism, ADHD, throat infections, well child visits, ED/UC reliance, and local PSG and tonsillectomy rates. Models were assessed for overdispersion using the deviance/degrees of freedom statistic. SAS v9 was used for all analyses. This study was approved by the Nationwide Children's Hospital Institutional Review Board.

## Results

### Subject Selection

We identified 1,009,007 Medicaid/CHIP beneficiaries ages 2 to 18 years with a diagnosis of oSDB in 2017 or 2018 from the US and District of Columbia (Figure 1). Slightly under half (438,396, 43.4%) of these beneficiaries were enrolled in the 15 states with high to moderate quality race‐ethnicity data for pediatric beneficiaries.^41^ A total of 33,769 (7.7%) were excluded due to unknown race‐ethnicity. Beneficiaries with race‐ethnicity other than Black, Hispanic, or White (18,229, 4.5%) were excluded because low oSDB case counts, ranging from 7992 (2.1%) for Asian to 1912 (0.5%) for Hawaiian and Pacific Islander. Finally, beneficiaries were excluded 68,125 (17.6%) for clinical history criteria; 13,553 (3.5%) for Medicaid enrollment criteria; and 298 (0.1%) for missing other demographic information. These exclusions resulted in an oSDB cohort of 304,415 children (Figure 1, Table 1 and Supplemental Table S2, available online).

**Table 1:** Study Subject Characteristics and Care Received, Obstructive Sleep Disordered Breathing, Medicaid Beneficiaries Ages 2‐18 Years, 2017‐2018, 15 States

		No further oSDB‐directed care	Care received (not mutually exclusive)		
	Total		Polysomnography	Otolaryngology	Tonsillectomy
Characteristic	N (%)	N (%)	N (%)	N (%)	N (%)
All beneficiaries	304,415 (100.0)	146,945 (100.0)	55,001 (100.0)	114,206 (100.0)	74,811 (100.0)
Race/Ethnicity					
Black non‐Hispanic	55,470 (18.2)	28,266 (19.2)	11,312 (20.6)	18,006 (15.8)	11,919 (15.9)
Hispanic	126,808 (41.7)	61,706 (42.0)	25,236 (45.9)	48,400 (42.4)	27,891 (37.3)
White non‐Hispanic	122,137 (40.1)	56,973 (38.8)	18,453 (33.6)	47,800 (41.9)	35,001 (46.8)
Urban/Rural^a^					
Urban	252,886 (83.1)	47,993 (87.3)	93,120 (81.5)	58,963 (78.8)	123,403 (84.0)
Rural	51,529 (16.9)	7008 (12.7)	21,086 (18.5)	15,848 (21.2)	23,542 (16.0)
Sex					
Female	147,157 (48.3)	70,483 (48.0)	24,817 (45.1)	56,421 (49.4)	38,258 (51.1)
Male	157,258 (51.7)	76,462 (52.0)	30,184 (54.9)	57,785 (50.6)	36,553 (48.9)
Age					
2 years	21,085 (6.9)	10,012 (6.8)	3245 (5.9)	8716 (7.6)	5243 (7.0)
3‐6 years	111,483 (36.6)	48,441 (33.0)	16,970 (30.9)	48,746 (42.7)	34,830 (46.6)
7‐12 years	112,717 (37.0)	55,378 (37.7)	21,450 (39.0)	41,329 (36.2)	26,356 (35.2)
13‐18 years	59,130 (19.4)	33,114 (22.5)	13,336 (24.2)	15,415 (13.5)	8382 (11.2)
Throat infections in past year					
None	183,957 (60.4)	95,306 (64.9)	37,648 (68.4)	60,584 (53.0)	35,415 (47.3)
1	65,436 (21.5)	31,223 (21.2)	10,504 (19.1)	25,883 (22.7)	17,732 (23.7)
2	30,387 (10.0)	12,587 (8.6)	4183 (7.6)	14,028 (12.3)	10,489 (14.0)
3‐4	24,635 (8.1)	7829 (5.3)	2666 (4.8)	13,711 (12.0)	11,175 (14.9)
Obesity					
Yes	22,899 (7.5)	9517 (6.5)	8636 (15.7)	7536 (6.6)	4172 (5.6)
No	281,516 (92.5)	137,428 (93.5)	46,365 (84.3)	106,670 (93.4)	70,639 (94.4)
Attention‐deficit/hyperactivity disorder					
Yes	38,300 (12.6)	22,966 (15.6)	8124 (14.8)	9213 (8.1)	5454 (7.3)
No	266,115 (87.4)	123,979 (84.4)	46,877 (85.2)	104,993 (91.9)	69,357 (92.7)
Asthma					
None	256,331 (84.2)	125,665 (85.5)	42,812 (77.8)	96,460 (84.5)	63,584 (85.0)
Mild or moderate	46,573 (15.3)	20,616 (14.0)	11,597 (21.1)	17,359 (15.2)	11,006 (14.7)
Severe	1511 (0.5)	664 (0.5)	592 (1.1)	387 (0.3)	221 (0.3)
Autism					
Yes	9886 (3.2)	5392 (3.7)	2530 (4.6)	2657 (2.3)	1354 (1.8)
No	294,529 (96.8)	141,553 (96.3)	52,471 (95.4)	111,549 (97.7)	73,457 (98.2)
Emergency department/urgent care reliance^b^					
Yes	38,913 (12.8)	20,440 (13.9)	4501 (8.2)	14,239 (12.5)	10,659 (14.2)
No	265,502 (87.2)	126,505 (86.1)	50,500 (91.8)	99,927 (87.5)	64,152 (85.8)
Well child visit					
Yes	191,154 (62.8)	89,615 (61.0)	35,529 (64.6)	75,433 (66.0)	48,597 (65.0)
No	113,261 (37.2)	57,330 (39.0)	19,472 (35.4)	38,773 (34.0)	26,214 (35.0)

^a^ Urban/rural based on RUCA 2010 for ZIP codes, (depts.washington.edu/uwruca/ruca‐uses.php).

^b^ Emergency department/urgent care reliance defined as 1/3 or more outpatient visits in these settings.

### Care Pathways

**Figure 2:**
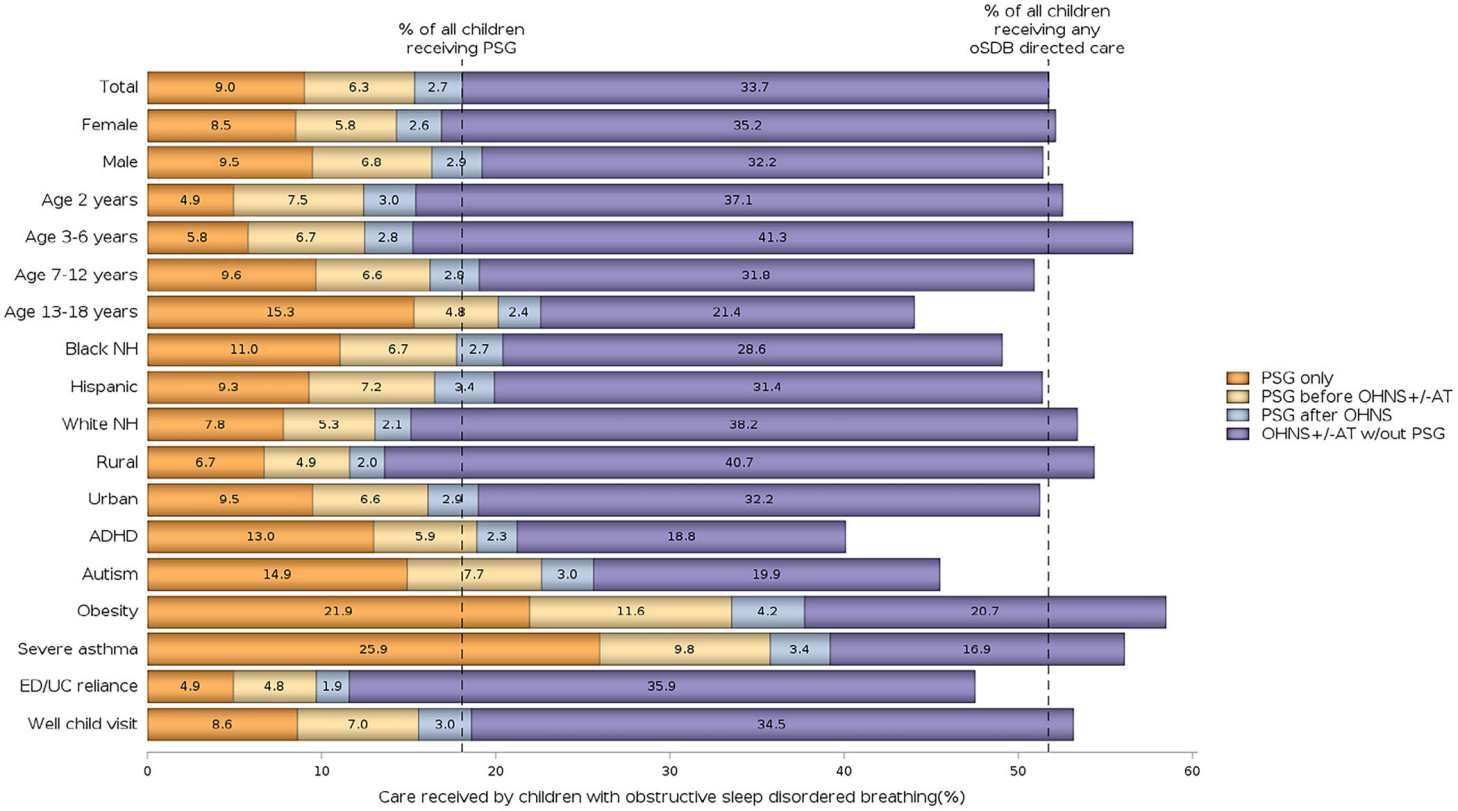
Care received for obstructive sleep disordered breathing by demographic and clinical characteristics, Medicaid beneficiaries ages 2 to 18 years, 2017 to 2018, 15 states. ADHD, Attention Deficit Hyperactivity; AT, Adenotonsillectomy; ED/UC, Emergency department/urgent care; NH, non‐Hispanic; OHNS, Otolaryngology, Head and Neck Surgery; PSG, polysomnography.

Approximately half (48.3%) of the oSDB cohort did not have evidence of oSDB‐directed care (Figures 1 and 2). Otolaryngology visit was the most frequent form of care (37.5%), followed by tonsillectomy (24.6%), and PSG (18.1%). Two‐thirds (66.1%) of the children who received PSG as their first form of care did not have any further care identified (Figure 1). Among children who saw an otolaryngologist, with or without PSG, 51.6% had a tonsillectomy. In contrast, among those who had PSG, with or without otolaryngology visit, 26.2% had a tonsillectomy. A small proportion of the cohort (4.1%) had tonsillectomy with no evidence of PSG or otolaryngology visit.

### Race and Ethnicity

The proportion of children with no evidence of oSDB‐directed care was higher among Black (51.0%) and Hispanic (48.7%) children relative to White (46.6%) children (*P* < .0001, Figure 2). The proportion of children who had a PSG, however, followed an inverse pattern, and was higher among Black (20.4%) and Hispanic (19.9%) children relative to White (15.1%, *P* < .0001).

These differences in care pathways were also evident after adjustment for age, sex, clinical factors, healthcare utilization, and local PSG and tonsillectomy rates (Table 2). Relative to White children, both Black and Hispanic children were 4% to 7% less likely to have otolaryngology visits and/or tonsillectomy but 6% more likely to have PSG (Table 2). This statistically significant higher likelihood of PSG was seen for PSG alone or in combination with other forms of care (Supplemental Table S3, available online), as well as regardless of the timing of PSG in relation to otolaryngology (Supplemental Table S4, available online).

**Table 2:** Rate Ratios and 95% Confidence Intervals by Type of Follow‐Up Care Relative to No Evidence of Care, Obstructive Sleep Disordered Breathing, Medicaid Beneficiaries Ages 2‐18 Years, 2017‐2018, 15 States

		Any oSDB‐directed care	PSG	OHNS	Tonsillectomy
Characteristic		RR [95% CI]^a^	RR [95% CI]	RR [95% CI]	RR [95% CI]
Race‐Ethnicity	Black	0.98 [0.97‐0.99]	1.06 [1.04‐1.09]	0.95 [0.93‐0.96]	0.95 [0.94‐0.97]
	Hispanic	0.97 [0.96‐0.98]	1.06 [1.04‐1.08]	0.96 [0.95‐0.97]	0.93 [0.91‐0.94]
	White	1 [Reference]	1 [Reference]	1 [Reference]	1 [Reference]
Urban/rural^b^	Rural	1 [Reference]	1 [Reference]	1 [Reference]	1 [Reference]
	Urban	1.01 [1.00‐1.02]	1.04 [1.01‐1.06]	1.01 [1.00‐1.02]	1.02 [1.00‐1.03]
Sex	Female	1 [Reference]	1 [Reference]	1 [Reference]	1 [Reference]
	Male	1.01 [1.00‐1.02]	1.07 [1.05‐1.08]	1.01 [1.00‐1.01]	0.98 [0.97‐0.99]
Age	2 years	1.21 [1.19‐1.23]	0.93 [0.90‐0.96]	1.44 [1.41‐1.47]	1.68 [1.63‐1.72]
	3‐6 years	1.28 [1.27‐1.30]	0.97 [0.95‐0.99]	1.53 [1.51‐1.55]	1.97 [1.93‐2.01]
	7‐12 years	1.17 [1.16‐1.19]	0.99 [0.97‐1.01]	1.35 [1.33‐1.37]	1.61 [1.57‐1.64]
	13‐18 years	1 [Reference]	1 [Reference]	1 [Reference]	1 [Reference]
Throat infection visits	None	1 [Reference]	1 [Reference]	1 [Reference]	1 [Reference]
	1	1.09 [1.08‐1.09]	0.95 [0.93‐0.96]	1.15 [1.14‐1.16]	1.28 [1.26‐1.30]
	2	1.22 [1.21‐1.23]	0.96 [0.94‐0.99]	1.34 [1.32‐1.35]	1.58 [1.56‐1.61]
	3‐4	1.42 [1.41‐1.44]	0.99 [0.96‐1.02]	1.60 [1.58‐1.62]	2.00 [1.97‐2.03]
Obesity	Yes	1.24 [1.22‐1.25]	1.74 [1.71‐1.77]	1.14 [1.12‐1.16]	1.14 [1.11‐1.17]
	No	1 [Reference]	1 [Reference]	1 [Reference]	1 [Reference]
ADHD	Yes	0.77 [0.76‐0.78]	0.95 [0.93‐0.97]	0.67 [0.66‐0.68]	0.59 [0.57‐0.60]
	No	1 [Reference]	1 [Reference]	1 [Reference]	1 [Reference]
Asthma	None	1 [Reference]	1 [Reference]	1 [Reference]	1 [Reference]
	Mild/Moderate	1.09 [1.08‐1.10]	1.34 [1.32‐1.36]	1.05 [1.04‐1.06]	1.04 [1.03‐1.06]
	Severe	1.14 [1.09‐1.19]	1.67 [1.57‐1.78]	0.91 [0.84‐0.99]	0.84 [0.75‐0.94]
Autism	Yes	0.93 [0.91‐0.96]	1.15 [1.11‐1.19]	0.84 [0.81‐0.86]	0.70 [0.67‐0.74]
	No	1 [Reference]	1 [Reference]	1 [Reference]	1 [Reference]
ED/UC reliance^c^	Yes	0.87 [0.86‐0.88]	0.69 [0.67‐0.71]	0.87 [0.86‐0.89]	0.91 [0.89‐0.92]
	No	1 [Reference]	1 [Reference]	1 [Reference]	1 [Reference]
Well child visit	Yes	1.03 [1.03‐1.04]	1.07 [1.05‐1.08]	1.06 [1.05‐1.07]	1.04 [1.03‐1.05]
	No	1 [Reference]	1 [Reference]	1 [Reference]	1 [Reference]
Local tonsillectomy rate (Quintile)^d^	1 (Low)	1 [Reference]	1 [Reference]	1 [Reference]	1 [Reference]
	2	1.04 [1.03‐1.06]	0.92 [0.89‐0.94]	1.07 [1.05‐1.09]	1.18 [1.15‐1.21]
	3	1.13 [1.11‐1.15]	0.93 [0.90‐0.96]	1.20 [1.17‐1.22]	1.49 [1.45‐1.54]
	4	1.19 [1.17‐1.21]	0.91 [0.88‐0.94]	1.29 [1.26‐1.32]	1.69 [1.63‐1.75]
	5 (High)	1.27 [1.24‐1.29]	0.88 [0.85‐0.92]	1.41 [1.38‐1.44]	1.94 [1.87‐2.01]
Local PSG rate (Quintile)	1 (Low)	1 [Reference]	1 [Reference]	1 [Reference]	1 [Reference]
	2	1.01 [1.00‐1.02]	1.41 [1.37‐1.46]	0.97 [0.96‐0.99]	0.96 [0.94‐0.98]
	3	1.05 [1.04‐1.06]	1.67 [1.62‐1.72]	1.01 [0.99‐1.02]	0.99 [0.98‐1.01]
	4	1.05 [1.03‐1.06]	2.01 [1.94‐2.08]	0.98 [0.96‐0.99]	0.95 [0.93‐0.97]
	5 (High)	1.08 [1.07‐1.10]	2.53 [2.45‐2.62]	0.97 [0.95‐0.98]	0.98 [0.96‐1.01]

Abbreviations: ADHD, Attention deficit/hyperactivity disorder; CI, confidence interval; OHNS, Otolaryngology, Head and Neck Surgery; PSG, polysomnography.

^a^ Rate ratio and 95% confidence intervals for care received relative to no evidence of PSG, OHNS, or AT based on Poisson regression, controlling for all variables shown in table and state of residence.

^b^ Urban/rural based on RUCA 2010 for ZIP codes, (depts.washington.edu/uwruca/ruca‐uses.php).

^c^ Emergency department/urgent care reliance defined as 1/3 or more outpatient visits in these settings.

^d^ Local rates are calculated as annual average rates from 2017‐2019 for all children enrolled in Medicaid, aggregated at the Census Public Use Microdata Area level.

### Urban/Rural

Children with ZIP Codes corresponding to urban residence had a higher proportion with no evidence of oSDB‐directed care (48.8%), relative to those with ZIP Codes corresponding to rural residence (45.7%, *P* < .0001, Figure 2). A higher proportion of children with urban residence also had PSG (19.0%) compared to those with rural residence (13.6%, *P* < .0001). After adjustment in the multivariable models, urban residence was associated with higher rates of care including PSG (adjusted RR = 1.04, 95% CI 1.01‐1.06, Table 2) and lower rates of care that did not include PSG (Supplemental Table S3, available online). Upon closer examination, urban children were more likely to have PSG alone or PSG prior to otolaryngology visits but less likely to have PSG after otolaryngology visits (Supplemental Table S4, available online).

### Race and Ethnicity in Combination With Urban/Rural

**Figure 3:**
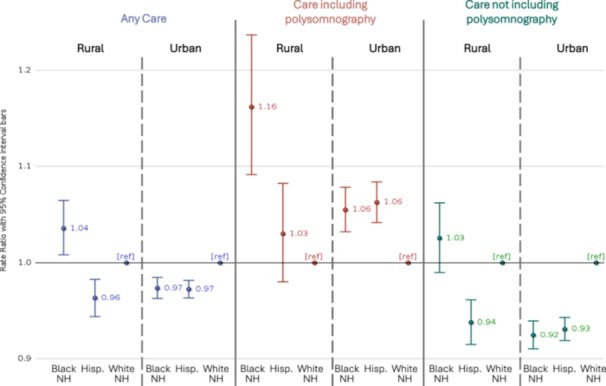
Combined effects of race‐ethnicity and urban/rural residence on care received for obstructive sleep disordered breathing, Medicaid beneficiaries ages 2 to 18 years, 2017 to 2018, 15 states. Rate ratio and 95% confidence intervals for care received relative to no evidence of polysomnography, otolaryngology, or tonsillectomy, controlling for demographics, medical conditions, and utilization, and local polysomnography and tonsillectomy rates. Hisp., Hispanic; NH, non‐Hispanic; ref, reference.

We observed statistically significant multiplicative interaction between urban/rural residence and Black relative to White race‐ethnicity for receipt of any form of care compared to no evidence of oSDB‐directed care (*P* < .0001); the RR for Black relative to White in the urban setting was 0.97 (95% confidence interval 0.96‐0.98) and for rural setting the RR was 1.04 (1.01‐1.06) (Figure 3). The statistically significant interaction was also evident for care without PSG (*P* < .0001), but not for care with PSG (*P* = .0554). We did not find any evidence of multiplicative interaction for urban/rural residence and Hispanic relative to White race‐ethnicity (Figure 3).

### Healthcare Utilization and Health History

Children who had a well child visit in the year prior to their oSDB index claim were slightly more likely to have any form of follow‐up care relative to children without a well child visit, although the association was weak (Figure 2, Table 2). Conversely, children who had ED/UC reliance were less likely to have received any oSDB‐directed care. These findings were consistent for all care pathways (Table 2, Supplemental Table S3, available online).

Several specific health conditions were associated with higher rates of PSG, including obesity, severe asthma, and autism (Figure 2, Table 2). Obesity was also associated with otolaryngology visits and tonsillectomy, although this appears to have been limited to these forms of care in combination with PSG, since obesity was not associated with otolaryngology and/or tonsillectomy without PSG (Supplemental Table S3, available online).

Children with ADHD were more likely to have PSG only but less likely to have other forms of oSDB‐directed care. The opposite was true for children with a history of throat infections, who were less likely to have PSG only and more likely to have care including otolaryngology visits and/or tonsillectomy.

### Local Rates of PSG and Tonsillectomy

Residence in an area with higher tonsillectomy rates was associated with higher rates of otolaryngology visits and higher rates of tonsillectomy, but lower rates of PSG (Table 2). Children living in areas with higher PSG rates were more likely to have PSG, but the rates of otolaryngology and/or tonsillectomy were only weakly associated with the local PSG rate. In models without adjustment for local rates, the RRs for race‐ethnicity and urban/rural were in the same direction but stronger than observed without adjustment (Supplemental Table S5, available online). For example, the RR for any PSG comparing urban residence to rural without controlling for local rate was 1.10 (1.07‐1.13) but decreased to 1.02 (0.99‐1.05) after controlling for the local rates. The RRs for the other exposures were not confounded by the local usage rates.

## Discussion

In this retrospective cohort study of Medicaid data, we observed Black and Hispanic children with an initial diagnosis of oSDB were less likely than White children to receive subsequent oSDB‐directed care. When stratified by type of subsequent care compared to no oSDB‐directed care, Black and Hispanic children were more likely to have PSG than White children, while White children were more likely to receive otolaryngology care and/or tonsillectomy without PSG. These patterns varied slightly by urban/rural residence. Rural Black children had the same rate of otolaryngology care and/or tonsillectomy without PSG as White children. While the overall effect sizes we observed are small for racial/ethnic differences in care, they may be considered clinically significant given the prevalence of oSDB.

The observations of lower rates of overall oSDB follow‐up care and elevated rates of PSG among children of color is consistent with prior reports.^11^, ^12^, ^26^, ^44^ In particular, the studies by Pecha^10^, ^11^ and Min^12^ also examined children with oSDB enrolled in Medicaid, using the MarketScan or state‐level databases. Our use of the T‐MSIS analytic files allowed us to use the ZIP Code of residence to derive local area estimates of PSG and tonsillectomy rates to control for geographic differences in access. The strength of the association between race‐ethnicity and type of care received decreased after controlling for local rates of polysomnography and tonsillectomy.

Unwarranted variation has been classified into 3 patterns: underuse of effective care (care that is well supported by evidence), variation in practice related to preference‐sensitive care (the evidence does not clearly support one option over another), and overuse of supply‐sensitive care (use of care is correlated with supply of care beyond clinical need).^36^ Care for pediatric oSDB is likely influenced by all 3 patterns. As with tonsillectomy, the “correct” rates of PSG among pediatric populations cannot be defined, so it is not possible to ascribe differences seen by race‐ethnicity and rurality to either over or underuse. What can be ruled out is that the differences reflect appropriate effective care based on clinical presentation, since Black and Hispanic children have a higher risk of OSAS and have more severe disease at presentation.^45^, ^46^, ^47^, ^48^, ^49^, ^50^, ^51^, ^52^ Higher OSAS risk and more severe disease would be indications for otolaryngology referral and tonsillectomy, the opposite of what we observed. Referrals for pre‐operative PSG for postoperative management would also not explain our results, since the majority of PSG was obtained prior to otolaryngology visits and was not followed by tonsillectomy.^37^

One possible explanation for higher PSG rates among children of color is that clinicians, aware of the higher risk of OSAS, are more likely to recommend PSG for children of color: an example of variation in preference‐sensitive care being influenced by professional opinion.^34^ Because it is not possible to determine indication for PSG using claims data, we cannot determine whether care pathways are in line with clinical guidelines.^37^ Individual risk factors, such as obesity and prematurity, also vary by race and ethnicity and may influence decisions to recommend PSG.^37^ Implicit racial bias may affect communication,^22^, ^53^, ^54^, ^55^, ^56^ but communication styles and recommendations may also vary appropriately based on clinician understandings of local cultural preferences as well as based on caregiver engagement.^56^, ^57^ For example, studies have shown Black parents are less likely to choose surgery over medical treatment options.^23^, ^53^ Clinicians, understanding their local communities and care preference patterns, might be more likely to suggest PSG for children of color because both published research and their own observations or assumptions regarding care preferences.

The unintended consequence of this example of culturally‐informed care is that PSG recommendations and referrals may be indirectly generating a pattern of underuse of subsequent care, including tonsillectomy, if PSG is associated with care delays and subsequent loss‐to‐follow‐up. Increased time to surgery as well as loss to follow‐up associated with PSG have also been reported in multiple single‐institution studies.^20^, ^24^, ^28^, ^58^ Caregiver reports of barriers to PSG include appointment availability, lack of care coordination, cost, and logistics.^59^, ^60^, ^61^

Controlling for local PSG and tonsillectomy rates made the estimated associations weaker for race‐ethnicity and for urban/rural residence, suggesting disparities in the use of these procedures could be sensitive to local supply, meaning children have different care pathways related to availability rather than clinical indication. Lack of PSG capacity, particularly in rural or underserved areas, has been an argument against use of routine PSG referrals for children identified as having oSDB in primary care.^62^ Supply‐sensitive variation in care creates inefficiencies in terms of cost, retention in care for children with oSDB, and lower capacity of PSG for children with other sleep‐related conditions.^62^

### Limitations

Use of claims data has inherent limitations related to data quality and completeness. Important to this study are that claims data only show receipt of polysomnography but not the test outcome, resulting in the inability to distinguish treatment intention from treatment received. As an example, among children who initially underwent PSG but had no further care identified, we cannot distinguish between children with findings of no sleep apnea who did not need further care and children who were subsequently referred to otolaryngology care but were lost to follow‐up.

Additionally, claims data can under ascertain potential confounders such as obesity. We were also unable to measure some forms of care such as encounters with sleep medicine specialty care because the difficulty of identifying these specialists in the Medicaid provider files. The primary data completeness concerns are related to missing race‐ethnicity for beneficiaries otherwise eligible for the study as well as the possibility of missed care either from incomplete coding of provider specialty or from unreported managed‐care encounter records. We also limited our analyses to 15 states due to poor‐quality data in the states we excluded. While this may have impacted generalizability, the 15 states included in our study account for 50% of the pediatric Medicaid population.

## Conclusion

We found that care pathways after diagnosis of oSDB differed substantially by race and ethnicity. Compared with White children, Black and Hispanic children had higher rates of no subsequent oSDB‐directed care. Black and Hispanic children had higher likelihood of PSG, but lower likelihood of specialty care and surgery without PSG. Most PSGs occurred before (or in the absence of) an otolaryngology evaluation. Prior studies indicate that undergoing PSG is associated with longer intervals to treatment and greater loss to follow‐up. Taken together, these findings suggest inequities in oSDB care reflect both underuse of effective treatments and differential use of preference‐sensitive and supply‐sensitive services. More judicious referral practices for PSG, coupled with stronger care‐coordination efforts to reduce loss‐to‐follow‐up, may help mitigate these disparities.

## Author Contributions

**Colleen C. McLaughlin**, **Jesse L. Hawke**, **Laura J. Chavez**, design, conduct, analysis, interpretation, presentation; **Emily F. Boss**, **Guy M. Brock**, **Meredith M. Lind**, **Tasleem J. Padamsee**, **Prasanth Pattisapu**, **Deena J. Chisolm**, design, interpretation, presentation.

## Disclosures

### Competing interests

None.

### Funding source

This work was supported by funding from the National Institute on Minority Health and Health Disparities R01MD016064 and the National Heart, Lung, and Blood Institute R01HL166504.

## Supporting information

Table S1 Code definitions for selection of study cohort and for categorization of exposures.
Table S2. Distribution of follow‐up care for children diagnosed with obstructive sleep disordered breathing
Table S3. Rate Ratios and 95% Confidence Intervals by type of follow‐up care relative to no evidence of care
Table S4. Rate Ratios and 95% Confidence Intervals by timing of polysomnography relative to otolaryngology visit, relative to no evidence of care
Table S5. Rate Ratios and 95% Confidence Intervals by type of follow‐up care relative ot no evidence of care without adjustment for regional tonsillectomy or polysomnography rates.

## Data Availability

Medicaid/CHIP data were obtained under Centers for Medicare and Medicaid Services Data Use Agreement # RSCH‐2022‐58522. Data cannot be shared by the authors.
